# Assessing the long-term safety and efficacy of COVID-19 vaccines

**DOI:** 10.1177/01410768211013437

**Published:** 2021-05-04

**Authors:** Azeem Majeed, Marisa Papaluca, Mariam Molokhia

**Affiliations:** 1 Department of Primary Care & Public Health, School of Public Health, Imperial College London, London W6 8RP, UK; 2 Pharmaceutical Regulatory Science and Innovation, Department of Public Health Sciences, King’s College London, London SE1 1UL, UK; 3 Clinical Epidemiology and Primary Care, King’s College London, London SE1 1UL, UK

Vaccines for COVID-19 were eagerly awaited, and their rapid development, testing, approval and implementation are a tremendous achievement by all: scientists, pharmaceutical companies, drugs regulators, politicians and healthcare professionals; and by the patients who have received them. But because these vaccines are new, we lack long-term data on their safety and efficacy.^1^ In surveys of people who define themselves as ‘vaccine hesitant’, this lack of long-term data is one of the main reasons given for their beliefs.^2^ Hence, providing this information is a public health priority and could help reassure vaccine-hesitant people that receiving a COVID-19 vaccine is the right choice for them.^3^ Emerging data from the UK and elsewhere are confirming the benefits of COVID-19 vaccines and this is one of the factors that is leading to a reduction in vaccine hesitancy in the UK population.

The news that two UK recipients of the COVID-19 Pfizer-BioNTech mRNA vaccine suffered severe allergic reactions on the first day of its rollout illustrates the need for accurate recording of any adverse events following administration of COVID-19 vaccines.^4^ These allergic reactions were unexpected and led to procedures being put in place to prevent further episodes of anaphylaxis after vaccine administration. Further concerns were raised after a high death rate was reported in elderly vaccine recipients in Norway.^5^ More recently, an association has been reported between the AstraZeneca COVID-19 vaccine and clotting disorders, which led to some countries restricting the use of the vaccine in younger people.

## Evidence from real-world studies

Early real-world data from vaccine recipients in England, Scotland and Israel show that vaccination provides a high level of protection from symptomatic COVID-19 infection and serious illness, along with a large reduction in the risk of hospital admissions.^6–8^ There is also now some evidence that COVID-19 vaccines can reduce the transmissibility of SARS-CoV-2.^9^ In a study from England, a single dose of either the Pfizer-BioNTech or AstraZeneca vaccine was around 80% effective at preventing hospitalisation in people aged 80 years and over, three to four weeks after vaccine administration.^6^ A Scottish study reported that the risk of hospitalisation was reduced by up to 85% after one dose of the Pfizer-BioNTech vaccine and up to 94% after one dose of the AstraZeneca vaccine, 28–34 days after vaccination.^7^ A study from Israel reported that the Pfizer-BioNTech vaccine was 94% effective at preventing symptomatic COVID-19 seven days after the second dose.^8^

## Data submission to medicines regulators

The studies submitted to medicines regulators (such as the Medicines and Healthcare products Regulatory Agency, US Food and Drug Administration, and the European Medicines Agency) for the initial approval of these vaccines only provided short-term data on vaccine efficacy; typically for less than four months after full vaccination. Therefore, the regulators – within the framework of authorisation both for their emergency use (UK and USA) or as conditional marketing authorisation (European Union) – required the pharmaceutical companies to ensure continuation of the ongoing clinical studies and the initiation of new ones, within specified timeframes, to fill information gaps identified by the regulators, mitigate risks, and refine knowledge of the safety aspects of the vaccines.^10–12^

In particular, in addition to established pharmacovigilance requirements of frequent safety reports (i.e. monthly safety update reports), reporting from ongoing and new studies were also imposed to the vaccine companies to establish and confirm the risks and benefits of the vaccines when used in special populations. Regulators required, for example, further data on vaccines in pregnant and breast-feeding women, immunocompromised patients, frail patients with co-morbidities, and patients with autoimmune or inflammatory disorders. Studies have also been required to address potential interaction with other vaccines, and provide long-term safety data.

In the context of the COVID-19 mass vaccination campaigns, it is imperative to promptly collect clinical data and outcomes in real-world settings. Real-world data coupled with relevant fundamental life sciences research such as the sequencing of strains of SARS-CoV-2 dominant in the population, and the in vitro challenging of immune system mechanisms, can provide independent information, essential for public health decision making.^13^ The data collection and real-time analyses are also important for evidence-based communications, and to re-assure the public that the UK and other countries have trusted and sustained ongoing scrutiny of the benefits and risks of the new COVID-19 vaccines. These data, for example, allowed pharmaceutical regulators across the world to review the evidence about the safety of the AstraZeneca COVID-19 vaccine and place limits on its use while awaiting further evidence.

## Addressing evidence gaps

Important questions remain to be answered from these ongoing reviews of vaccine safety and efficacy. For example, we do not yet have information on how long the immunity generated by COVID-19 vaccines will last, or on whether and how well they will protect against new variants of SARS-CoV-2. Longitudinal data on ‘vaccine failures’, or re-infections for example, can help guide national policies on how frequently booster doses of vaccine are needed to maintain a good level of immunity in the population, and on whether vaccines need modification to provide protection against new variants of SARS-CoV-2. In addition, it will be essential to identify COVID-19 clusters and potential variants emerging under vaccination pressure in clinical settings, to enact the appropriate public health protection measures. This will require considerably longer-term follow-up than we have seen in clinical trials and real-world evaluations thus far.

The UK is well-placed to collect these data and to secure its timely evaluation and integration with information provided by its strong life sciences research industry, to guide public health decision making, as the long-term efficacy and safety of vaccines encompass not only effects on recipients but also the overall effects on the COVID-19 pandemic. We also have a National Health Service that has developed computerised medical records for use in general practices on a population of around 67 million people.^14,15^ These electronic medical records provide longitudinal data on people’s health and medical experiences and can be used to monitor vaccine uptake by population group. Early results from UK studies show a lower uptake of COVID-19 vaccines among people from ethnic minority groups and people living in poorer parts of the UK.^16^

Data from electronic medical records can now also be linked to other data, such as hospital admissions records and mortality records, as well as to the results of COVID-19 tests, increasing their value for monitoring the safety and efficacy of the new COVID-19 vaccines. The UK is also has well-established programmes for the genomic sequencing of SARS-CoV-2.^17^ The comprehensive nature of these National Health Service medical records and the large population they cover mean that they can be used to look at safety and efficacy of COVID-19 vaccines in specific populations. This could be, for example, by age, sex, medical history or ethnic group. It would also be possible to look at more serious health outcomes and death rates by linkage to other datasets. A key step in providing this information in the UK and globally is to accurately record vaccination status (including the specific vaccine given) and any subsequent events in electronic medical records, as well reporting any adverse events to national medicines regulators such as the Medicines and Healthcare products Regulatory Agency in the UK. It is also important that the protocols for such studies and their findings are placed in the public domain so that they can be easily accessed by the public and by health professionals.

The usefulness of these real-world data will be facilitated by the recently developed standardised clinical codes for COVID-19 vaccines to record information in electronic medical records. These include, for example, codes for whether people attended or did not attend for their vaccination appointment, whether they declined to be vaccinated, and whether they had a clinical contra indication to being vaccinated. Other codes allow recording of the specific vaccine that was administered, which will be essential for comparing the long-term safety and efficacy of different COVID-19 vaccines.

## Reporting of adverse events

The data from electronic medical records can be supplemented by the reporting of any suspected adverse events by health professionals or members of the public to national medicines regulators, such as the Medicines and Healthcare products Regulatory Agency in the UK via the Yellow Card Scheme. Vaccine recipients should also be encouraged to report any reactions directly to the medicines regulators as well as to their doctor. This allows medicines regulators to compile information on the safety profile of the new COVID-19 vaccines (which are now issued regularly) and advise patients and the public of any potential problems.^18^ The Medicines and Healthcare products Regulatory Agency is also developing an artificial intelligence tool to evaluate reports of adverse reactions to COVID-19 vaccines.^19^ There are further initiatives to monitor adverse effects from COVID-19 vaccines in the European Union led by the European Medicines Agency.^20^

## Conclusions

As long-term data on the safety and efficacy build globally, these can address many of the concerns that vaccine-hesitant people have about COVID-19 vaccines, thereby creating a positive environment that encourages higher uptake of vaccination. These data will also guide national public health policies, such as how frequently to provide booster doses of vaccine and whether limits should be placed on the use of a specific vaccine.

Vaccination remains the best way to control the COVID-19 pandemic, and countries globally should work together to generate the information needed to provide long-term data on safety and outcomes. Because of the very rare nature of some side effects, this will require international collaboration so that data from countries can be pooled to allow more precise estimates of risk to be calculated. This will include using data from low- and middle-income countries once vaccination programmes are established there, as well as from marginalised groups in higher-income countries, to ensure that the data are fully representative of the global population.^21^

## Declarations

**Ethics approval:** Not required.

**Guarantor:** The guarantor is AM.

**Contributorship:** AM, MM and MP wrote the article.

**Provenance:** Not commissioned; peer-reviewed by Neil Snowsie and Gayle DeLong.
